# Artificial Neural Network and Response Surface-Based Combined Approach to Optimize the Oil Content of *Ocimum basilicum* var. *thyrsiflora* (Thai Basil)

**DOI:** 10.3390/plants12091776

**Published:** 2023-04-26

**Authors:** Akankshya Sahu, Gayatree Nayak, Sanat Kumar Bhuyan, Abdul Akbar, Ruchi Bhuyan, Dattatreya Kar, Ananya Kuanar

**Affiliations:** 1 Centre for Biotechnology, Siksha ‘O’ Anusandhan University, Kalinga Nagar, Ghatikia, Bhubaneswar 751003, Odisha, India; 2 Institute of Dental Sciences, Siksha ‘O’ Anusandhan University, Bhubaneswar 751003, Odisha, India; 3 Department of Biotechnology, Odisha University of Technology & Research, Bhubaneswar 751003, Odisha, India; 4 Department of Medical Research, Health Science, IMS & SUM Hospital, Siksha ‘O’ Anusandhan University, Bhubaneswar 751003, Odisha, India

**Keywords:** Thai basil, essential oil, artificial neural network, climatic factors, soil parameters

## Abstract

*Ocimum basilicum* var. *thyrsiflora* is valuable for its medicinal properties. The barriers to the commercialization of essential oil are the lack of requisite high oil-containing genotypes and variations in the quantity and quality of essential oils in different geographic areas. Thai basil’s essential oil content is significantly influenced by soil and environmental factors. To optimize and predict the essential oil yield of Thai basil in various agroclimatic regions, the current study was conducted. The 93 datasets used to construct the model were collected from samples taken across 10 different agroclimatic regions of Odisha. Climate variables, soil parameters, and oil content were used to train the Artificial Neural Network (ANN) model. The outcome showed that a multilayer feed-forward neural network with an R squared value of 0.95 was the most suitable model. To understand how the variables interact and to determine the optimum value of each variable for the greatest response, the response surface curves were plotted. Garson’s algorithm was used to discover the influential predictors. Soil potassium content was found to have a very strong influence on responses, followed by maximum relative humidity and average rainfall, respectively. The study reveals that by adjusting the changeable parameters for high commercial significance, the ANN-based prediction model with the response surface methodology technique is a new and promising way to estimate the oil yield at a new site and maximize the essential oil yield at a particular region. To our knowledge, this is the first report on an ANN-based prediction model for *Ocimum basilicum* var. *thyrsiflora*.

## 1. Introduction

*Ocimum basilicum* var. *thyrsiflora* (Thai basil) is an important industrial medicinal plant belonging to the *Ocimum* species of the Lamiaceae family, which can be used in both raw and processed forms in traditional medicine and the pharmaceutical industry [1]. *Ocimum* species grow well in saline and alkaline soils with a moderately acidic pH, moderate to heavy rainfall, normal humidity, and high temperatures. This plant is mainly grown in tropical Asia, Africa, and Central and South America, although it is especially popular in China, Japan, Turkey, and Iran [2]. *Ocimum* species are well recognized for their essential oil, which is responsible for condiment flavour and plant aroma [3]. Thai basil oil has a significant commercial value because of the presence of phenylpropanoids such asestragole, methyl eugenol, (E)-α-bergamotene, and their derivatives, and terpenoids such as monoterpene alcohol linalool, limonene, and terpinolene. The most prevalent volatile components identified in the aroma profile of dried Thai basil included estragol, methyl eugenol, and (E)-α-bergamotene [4]. Over the past few decades, extensive research has shown that *Ocimum basilicum* extracts have antimicrobials, antioxidants [3], antiviral properties [5], anti-inflammatory properties [6], hypolipidemic effects [7], anti-platelet aggregation, and antithrombotic, antiulcerogenic, and anticarcinogenic [8] activities. It can decrease blood levels of LDL cholesterol while raising blood levels of HDL cholesterol, hence reducing cardiovascular diseases [9,10]. The export database reveals that India exported 1553 Rs per Kg of basil essential oil during 2016–2017 [11].

The major limitation in the commercial production of Thai basil is the lack of staging of high essential oil-containing genotypes and variation in the oil content in different agroclimatic regions. Because the production of essential oils is primarily driven by environmental conditions, it would be impossible to identify the genetically superior Thai basil with a high essential oil concentration using a simple chemotype. According to Rawat et al., there is a wide range of variations in the essential oil concentration of *Ocimum* species in various agroclimatic areas of Uttarakhand [12]. To ascertain the link between biochemical content and environmental variables, many statistical techniques are used. Multiple linear regression (MLR) analysis and correlation are two common statistical procedures that can only be used to find linear associations and are ineffective when applied to non-linear data [13]. Artificial neural networks (ANN) are increasingly routinely used to build and map non-linear relationships between inputs and outputs due to their improved prediction accuracy. ANN modelling is used to simulate how the human brain functions [14]. It was selected because it can quickly pick up lessons from events without first running statistical analyses on the parameters [15]. The input layer, hidden layers, and output layer of neurons are the three main divisions of an ANN [16]. The input layers’ neurons receive the input data, which is then adjusted before being sent to the hidden layer [17]. Each neuron in the layer below performs a linear combination of the data from the neurons in the input layer, adding weight values related to certain nodes to the result. The projected model is the outcome of the neurons in the hidden layer combining the linear data from the input layer with a transfer function (a specific non-linear function) [16]. The ANN model has been used to predict the bioactive content of the compounds podophyllotoxin from Podophyllum hexandrum [18]; hyperforin, hypericin, and pseudohypericin from *Hypericum perforatum* L. [13]; and bacoside A from Bacopa monnieri [19].

Therefore, it will be mandatory to analyze the soil parameters and climatic factors of different agroclimatic areas of Odisha for high essential oil content. An Artificial Network (ANN) model and a Response surface-based combined approach for essential oil content in Thai basil can be developed to predict the appropriate site and enhance the essential oil content at a particular site by managing the sensitive and variable factors.

## 2. Materials and Methods

### 2.1. Plant Materials and Sample Station

From June to October 2021, samples of Thai basil leaves were collected from 93 sites across 10 agroclimatic regions in several districts of Odisha, at varying altitudes ranging from 0.1 to 1204 m (Table 1). Three replicates of the leaf samples were taken from each site. The two duplicates were separated by 2 to 5 m. To get rid of the dust, the samples of freshly collected leaves were first washed with running tap water and then with distilled water. Following air drying at room temperature, samples of washed leaves were used to determine the essential oil contents. To analyze soil nutrients, triplicate soil samples were taken from each sampling site and brought to the lab. The documented monthly average data on environmental variables, such as rainfall, temperature, or humidity, were noted from each sampling site from June 2021 to October 2021.

### 2.2. Extraction of Essential Oil and Quantification

The air-dried leaves were crushed and powdered in a mortar and pestle before being hydrodistilled in a Clevenger-type apparatus made entirely out of glass. For later usage, the oil was dried with anhydrous sodium sulphate and stored in a sealed Eppendorf tube at 4 °C.

### 2.3. Quantitative Analysis of Soil

In triplicate, soil samples were taken from each sampling site within an agroclimatic region. A soil sample of approximately 250 g was taken and sieved through a 2-mm mesh. Nutrient analysis was performed using fine soil. Using the Systronics pH meter (Model MKVI), the pH of soil samples was identified in soil: water 1:2 ratio suspension after 30 min of equilibration with infrequent stirring.

Using Bray’s No 1 technique, the total phosphorus content of soil samples was determined. The solution was made by extracting 2 g of soil in 40 mL of Bray’s solution, which contains 0.03 NH_4_F and 0.025 N HCL. This mixture was then agitated vigorously for five minutes using a mechanical shaker and filtered through Whatman paper. A 25 mL flask was filled with a 0.5 mL aliquot. Ammonium molybdate solution (0.5 mL) and distilled water were added to bring the volume up to 25 mL. To make up the volume, diluted SnCl_2_ (0.5 mL was diluted in 66 mL) was added. The concentration of phosphorus was measured using a spectrophotometer (Model: Systronics 166) set at 660 nm. The concentration was measured using a standard graph created by varying the phosphorus content. The available phosphorus in the soil samples was determined by extracting the soil using Olsen’s reagent (0.5 M NaHCO_3_, pH 8.5). The phosphorous concentration was measured using a spectrophotometer (Model: Systronics 166) and the mechanism was ascorbic acid reduced to blue-colored sulphomolybdic acid in the sulphuric acid system at 882 nm [20]. Next, 5 g of the soil sample was placed in a 100 mL conical flask along with 25 mL of 1 N NH_4_OAc solution to determine the amount of potassium present in the soil. An automatic shaker was used to shake the mixture for 5 min, after which the filtrate’s potassium level was determined using a flame photometer (Model: Systronics 128). The Walkley and Black Wet Digestion Technique were used to calculate the organic carbon content in the soil sample by chemical analysis [21].

The alkaline KMnO_4_ method was used to calculate nitrogen content. Next, 20 g of the soil sample were placed in an 800 mL Kjeldahl flask together with 100 mL of a 0.32% KMnO_4_ solution, 2.5% NaOH solution, and distilled water. In a 250 mL conical flask containing 20 mL of 2% boric acid and a mixed indicator, the distillation process was continued, and the result was collected in a receiver tube. The available nitrogen was then calculated by titrating the distillate in a burette against 0.02 N H_2_SO_4_ to a pink colour endpoint [22].

### 2.4. Data Exploration

All computational work (model development, plot generation, etc.) was performed using R (R Core Team 2021, Vienna, Austria). The data set consists of 12 features and 93 instances. Out of the features, 11 are predictors. The predictors are phosphorous, nitrogen, potassium, the organic carbon content, pH of the soil, maximum and minimum relative humidity, average rainfall, maximum and minimum average temperature, and altitude. Thai basil oil content is the response. Standard deviations for all features were calculated using the mlbench [23] library (Appendix A).

The formula for standard deviation is provided below.

(1) $$\mathrm{Standard}\;\mathrm{deviation}=\sqrt{\frac{{\left(x-\overset{-}{x}\right)}^{2}}{n-1}}$$

where *x* and $\overset{-}{x}$ are the value of each observation and mean of all observations, respectively.

**Figure 1:**
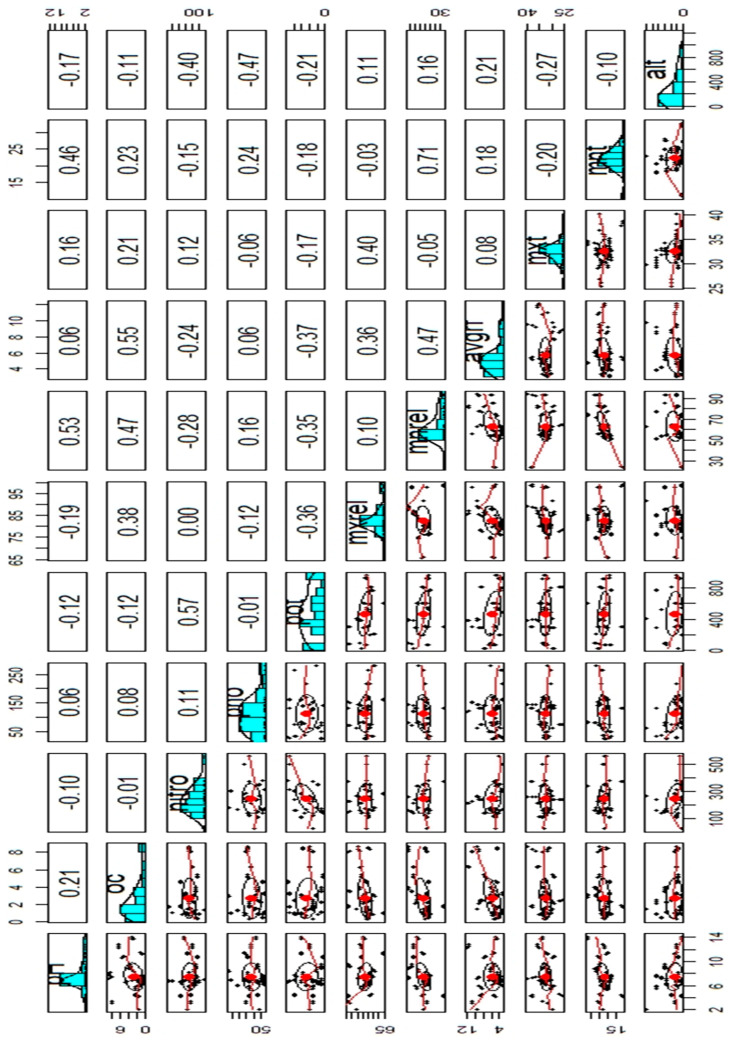
Panel plot to investigate the interaction of predictors with the response.

Pearson’s correlation coefficient between features and data distribution in each feature were evaluated using the package psych [24]. Pearson’s correlation coefficient was calculated using equation 2. The correlation values were provided in the panel plot in the form of numeric values as well as a correlation ellipse (Figure 1).

(2) $$\mathrm{The}\;\mathrm{Pearson}’s\;\mathrm{Correlation}\;\mathrm{coefficient}\;(r)=\frac{\sum \left\{(x-\overset{-}{x})(y-\overset{-}{y})\right\}}{\sqrt{\sum {\left(x-\overset{-}{x}\right)}^{2}\sum {\left(y-\overset{-}{y}\right)}^{2}}}$$

where *x* and *y* are the values of the two variables; $\overset{-}{x}\;\mathrm{and}\;\overset{-}{y}$ are the respective means.

### 2.5. Data Splitting

The dataset (total data) is divided into three sets: train, test, and validation with 70%, 20%, and 10% of data, respectively. The train set was used to develop the model by training. The model was evaluated using the test set. Finally, the model was validated using a validation set.

### 2.6. Artificial Neural Network Model Development

The Caret (classification and regression training) package [25] was used to develop the artificial neural network model. Data was scaled using minimum–maximum normalization. The train set was resampled with 15-fold cross-validation during training. A grid-tuning approach was used to ascertain the optimal number of layers and nodes within each layer. The logistic function was selected as the activation function. A learning rate of 0.02 was maintained. Error calculations were performed using the Sum of Squared Errors.

(3) $$\mathrm{SSE}=\sum {\left(y-\hat{y}\right)}^{2}$$

where *y* and $\hat{y}$ are the actual response and predicted response, respectively.

### 2.7. Model Evaluation and Selection

The final model was selected based on the values of root mean square error (RMSE), mean absolute error (MAE), and the coefficient of determination or R squared (R squared) values. The above evaluation metrics were calculated using the following formulae:

(4) $$\mathrm{RMSE}=\sqrt{\frac{\sum {\left(\hat{y}-y\right)}^{2}}{n}}$$

(5) $$\mathrm{MAE}=\frac{\sum \left(y-\hat{y}\right)}{n}$$

(6) $$R\;\mathrm{squared}=\frac{\sum {\left(\hat{y}-\overset{-}{y}\right)}^{2}}{\sum {\left(y-\overset{-}{y}\right)}^{2}}$$

### 2.8. Variable Importance

The variable (different factors) importance of the response was evaluated using the Neural Net Tools [26] library. To determine the ranking importance of predictors, Olden’s method was used. To assess the significance of the predictors, the method analyzes the raw connection weights between input-hidden nodes and hidden-output nodes [27]. They have also compared several methods viz. input perturbation, partial derivatives, etc., and concluded that the connection weight approach—i.e., Olden’s method—is more reliable.

### 2.9. Partial Dependence Plots and Faceted Heatmap

Partial dependence plots (PDP) were generated to investigate the interaction of predictors with the response. These plots were generated using the PDP library. The use of linear plots is not suitable for explaining the complex relationship of different variables with the response. PDP plots are used to interpret the output of complex machine-learning models [28]. In this study, single-variable and multiple-variable PDPs are generated. Smoothing is applied using locally weighted regression (LOESS) in the case of single variable PDPs, which has popularity in the smoothing of scatterplots [29]. LOESS can perform well even if the response is a nonlinear function of the predictor [30]. The relationship of the response variable with two predictors is represented by a two-dimensional contour and three-dimensional PDPs.

Another type of plot called a faceted heatmap was used to identify the behavior of the response concerning precise value ranges of predictors. For this purpose, a lime [31] package was used.

### 2.10. Sensitivity Analysis

It is critical to identify the most important elements influencing essential oil content. Therefore, Garson’s algorithm was used to discover the influential predictors. The link strengths between the nodes are calculated to assess the relative importance of each predictor.

## 3. Results and Discussion

The pair plots (Figure 1) show four different data set representations through ellipse plots, scatter plots, correlation coefficients, and histograms. The trend lines represent the linear relationship among variables in scatter plots. The correlation ellipse characterizes the correlation strength. When the stretch is higher, the correlation coefficient is also higher. When the correlation coefficient value is between 0 to 0.1, 0.1 to 0.39, and 0.4 to 0.69, the correlation is negligible, weak, or moderate, respectively. Correlation is strong when the coefficient value is between 0.7 and 0.89, and very strong when the correlation coefficient value is between 0.9 and 1 [32]. From the plot, pH has a moderate correlation with variables viz. minimum relative humidity and minimum average temperature. Correlations of pH with remaining variables were found to be negligible to weak. Similarly, the correlation strengths among variables are represented in Figure 1. Only two variables, minimum relative humidity and minimum average temperature, have shown a strong correlation, i.e., 0.71. A correlation study among the predictors has significance in model evaluation. The machine learning algorithms are affected if any correlation exists among the predictors [33]. The data set shows multicollinearity among its variables. In such cases, artificial neural networks (ANN) are found to be suitable as the ANN models are least affected due to correlations among input variables [34,35].

### 3.1. Model Evaluation and Selection

**Figure 2:**
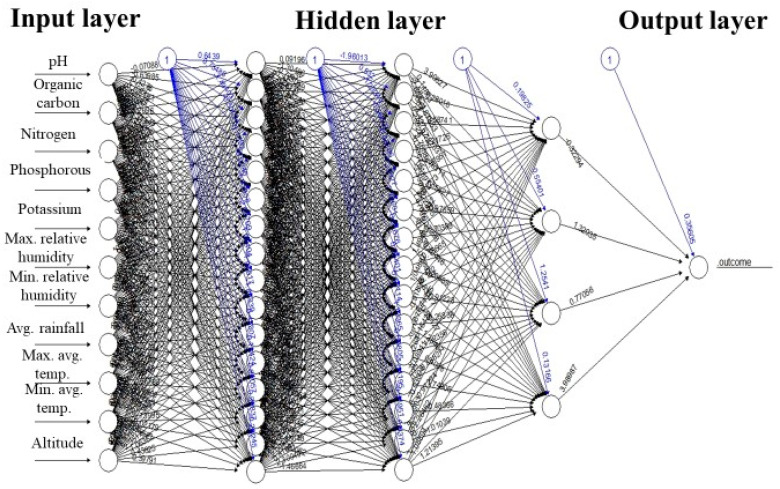
The selected resilient backpropagation ANN model with weight bracketing with three hidden layers, bias, and connection strengths.

**Figure 3:**
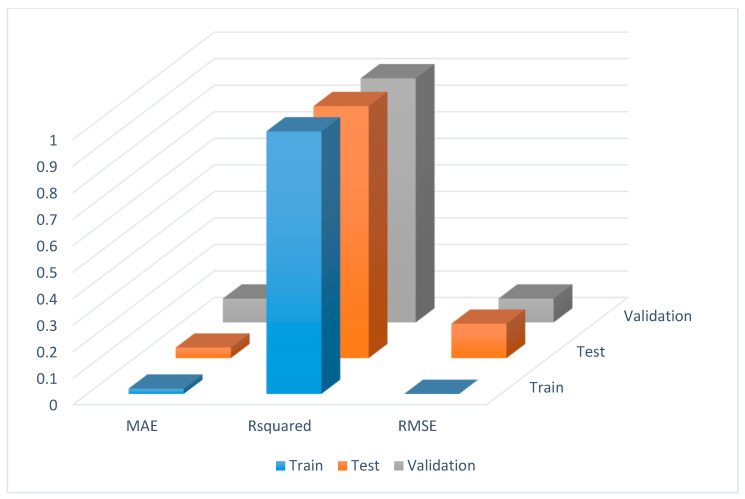
Mean absolute error (MAE), R squared, and Root mean squared error (RMSE) for train, test, and validation data.

The final model along with climatic data (Table 2), soil data (Table 3), different layers, nodes, and connection strengths are provided in Figure 2. Root mean square error (RMSE) and mean absolute error (MAE) were used as performance measures to select the best model. The data set has multicollinearity among variables, so the RMSE can be used as a performance measure [35]. The model that had the lowest RMSE and MAE was selected. The RMSE, mean absolute error (MAE), and the coefficient of determination (R-squared) values of the model for the train, test, and validation data sets are provided in Figure 3. The predictions and the actual responses for the train set are provided in Table 4. The RMSE, MAE, and R squared values for training were 0.00, 0.02, and 0.99, respectively (Figure 3).

After training, the model was evaluated using the test data set. The RMSE, MAE, and R squared values for test set data are 0.04, 0.13, and 0.95, respectively (Figure 3). Furthermore, the model was validated with a validation set. The model also performed well with RMSE, MAE, and R squared values of 0.09, 0.09, and 0.92, respectively. Table 5 and Table 6 show the predictions and actual responses for test and validation data, respectively. As far as we know, this is the first study to represent the extent of the essential oil content prediction of Thai basil. For predicting the essential oil content of Thai basil, the Artificial Neural Network (ANN) is recommended as one of the most promising methods. This networking system provides new possible approaches for the study of bioactive compounds in other plants as well as in other environmental conditions. As a predictive approach for maximizing the operating parameters during the extraction of various natural products, the ANN has been proposed by various researchers [36,37,38,39].

ANN does not require any inference of previous data structure; thus, it gathers an advantage over other statistical modeling techniques. It may detect complicated interactions and non-linear correlation, and expose the unknown linkage between previously input parameters [40]. The four statistical quality measures such as coefficient of determination (R squared), root mean square error (RMSE), mean absolute error (MAE), and mean absolute percentage error (MAPE) are used to develop the ANN model. When the coefficient value is 0.9–1, the correlation is very strong, and 0.7–0.89 indicates a strong correlation. The correlation is negligible when the correlation coefficient value is between 0–0.1; likewise, 0–1–0.39 and 0.4–0.69 indicate that the correlation is weak and moderate, respectively [32]. In model evaluation, the correlation study among predictors has significance. If any correlation is present between the predictors, then the machine learning algorithms are affected [33]. The ANN model developed in this study determined the strong predictive potential for essential oil content for Thai basil because it was measured by correlation coefficient (R squared) and root mean square error (RMSE). Less variation between the projected value and experimental value is indicated by the model’s higher R squared value of 0.92 and lower RMSE value of 0.09. The ANN model is called stronger when the R squared value is closer to 1 and the RMSE value is lower. Hence, it is possible to conclude that the model developed for the prediction of the essential oil content of Thai basil is considerably accurate. Similar studies were published in which error values were lower with high predictive analysis of the ANN model [41,42,43].

### 3.2. Significant Predictor Identification

**Figure 4:**
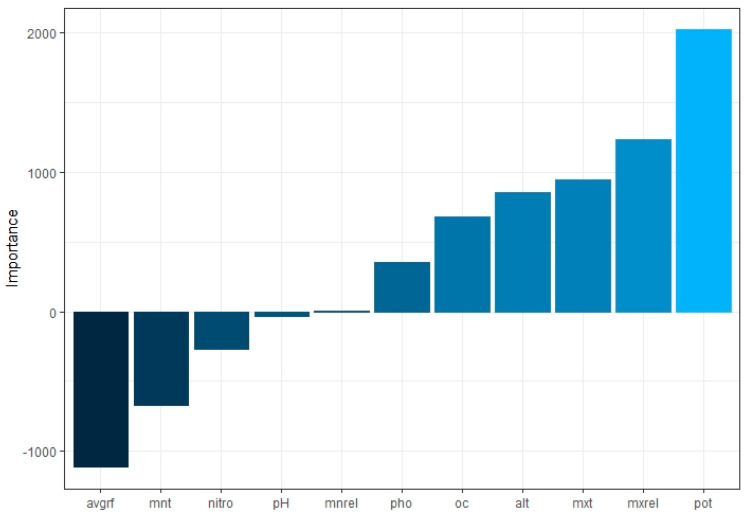
Relative importance of predictors.

According to the model, soil potassium content was found to have a very strong influence on response, followed by maximum relative humidity and average rainfall, respectively (Table 7). Minimum relative humidity was found to have the least effect on oil content. The relative importance of all variables on output is provided in Figure 4. Garson’s algorithm was used to discern influential predictors and remove insignificant predictors. In an ANN, the coefficient in a generalized linear model is partially equivalent to the weights that connect neurons. There are different weights connecting one predictor to the outcome, and the weights’ combined influence shows how important each predictor is about the outcome variable. A large number of adjustable weights makes an ANN very flexible and nonlinear, but also creates difficulties in interpretation [44,45]. A single value ranging from 0 to 1 that illustrates the relative relevance of predictors is produced by combining and scaling all weights that are linked to a predictor. The Neural Networking Tools (Version 1.5.1) package in R can be used to determine the relative importance [17].

### 3.3. Effect of Individual Predictors on Essential Oil Content

**Figure 5:**
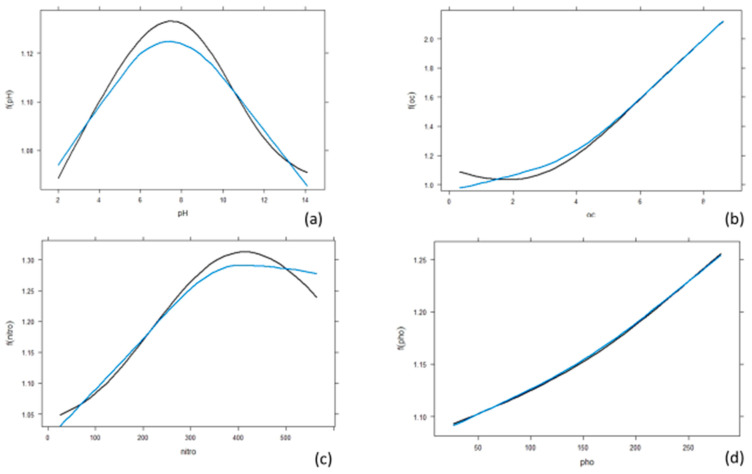

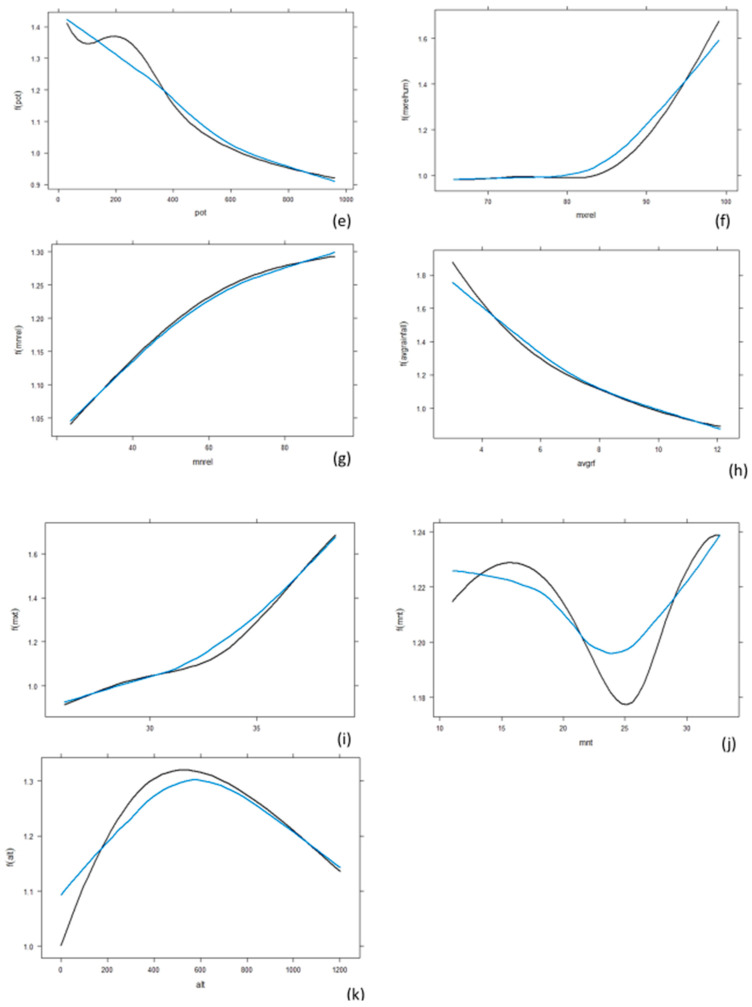
(**a**) Partial dependence plot of response in terms of pH; (**b**) partial dependence plot of response in terms of organic carbon; (**c**) partial dependence plot of response in terms of nitrogen; (**d**) partial dependence plot of response in terms of phosphorous; (**e**) partial dependence plot of response in terms of potassium; (**f**) partial dependence plot of response in terms of maximum relative humidity; (**g**) partial dependence plot of response in terms of minimum relative humidity; (**h**) partial dependence plot of response in terms of average rainfall; (**i**) partial dependence plot of response in terms of maximum average temperature; (**j**) partial dependence plot of response in terms of minimum average temperature; (**k**) partial dependence plot of response in terms of altitude. Black color line is for response and blue color line is for different factor.

**Figure 6:**
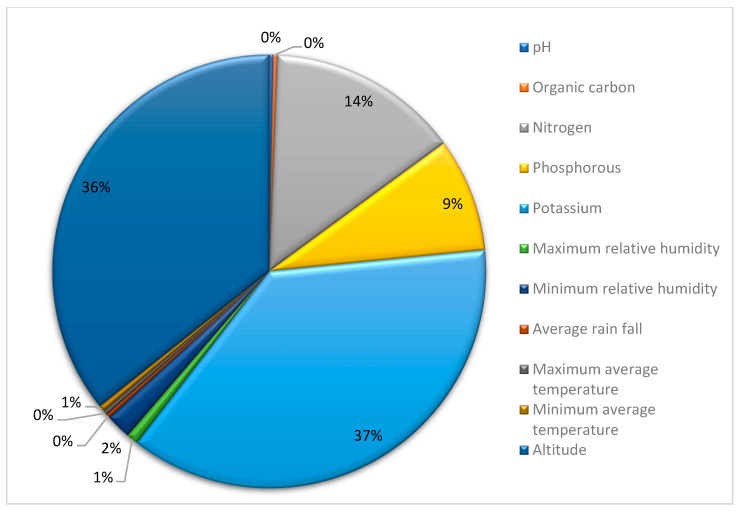
Sensitivity analysis of input parameters on oil yield (output).

Single variable PDPs are generated for all predictors and provided in Figure 5a–k. The multicollinearity among the features of the data set is shown in Figure 1. Most of the correlation among variables is weak to moderate. Minimum average temperature and minimum average relative humidity have shown a strong correlation (Figure 6). In such cases, ANN models provide promising results and are free from any biases due to multicollinearity [34]. Therefore, PDPs generated through artificial neural network models are suitable to study the change in response to predictors. The variation of oil content with soil potassium content was provided in Figure 5e. The essential oil content was found to be higher with low soil potassium content and average rainfall (Figure 5h). However, the oil content increased with an increase in organic carbon content (Figure 5b), nitrogen (Figure 5c), maximum average temperature (Figure 5i), maximum relative humidity (Figure 5f), etc. The oil content showed dramatic variation with variation in minimum average temperature (Figure 5j). Initially, the oil content was found to decrease with an increase in minimum average temperature. When the temperature is beyond 25 °C, the oil content gradually increased. Similarly, when the maximum relative humidity exceeded 85 (approx.), the oil yield also gradually increased (Figure 5f). Partial dependence plots (PDPs) were used to show how one or two variables affect the predictions of the model. In this study, a univariate partial dependence plot is displayed alongside each pairwise partial dependence plot in a matrix-style layout. An analyst can easily observe and identify the important pairs of variables that have a significant impact on the model using this partial dependence plot.

### 3.4. Mutual Effect of Two Predictors on Response

**Figure 7:**
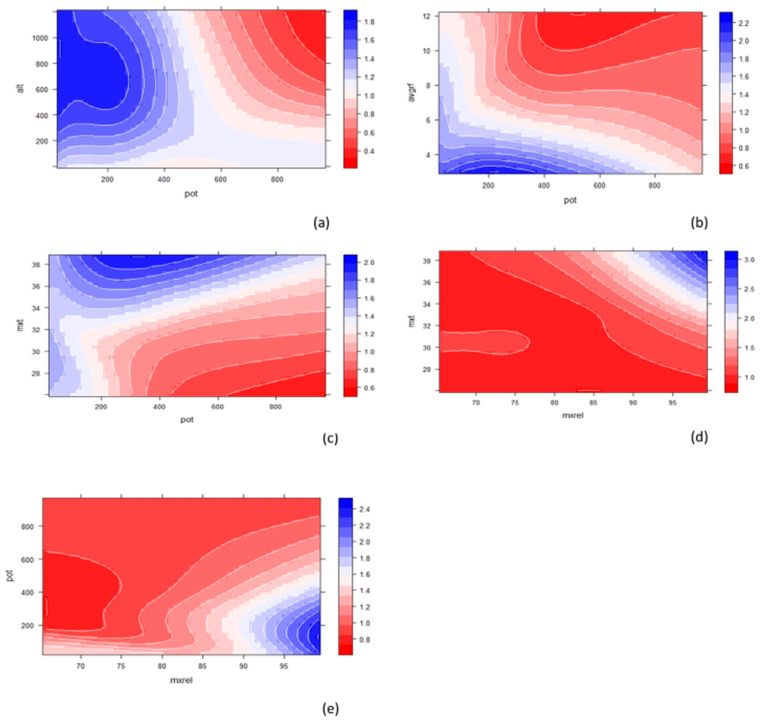
(**a**) Contour plot of soil potassium content, altitude, and essential oil content; (**b**) contour plot of soil potassium content, average rainfall, and essential oil content; (**c**) contour plot of soil potassium content, average temperature, and essential oil content; (**d**) contour plot of soil potassium content, average maximum relative humidity, and essential oil content; (**e**) contour plot of soil potassium content, pH, and essential oil content.

**Figure 8:**
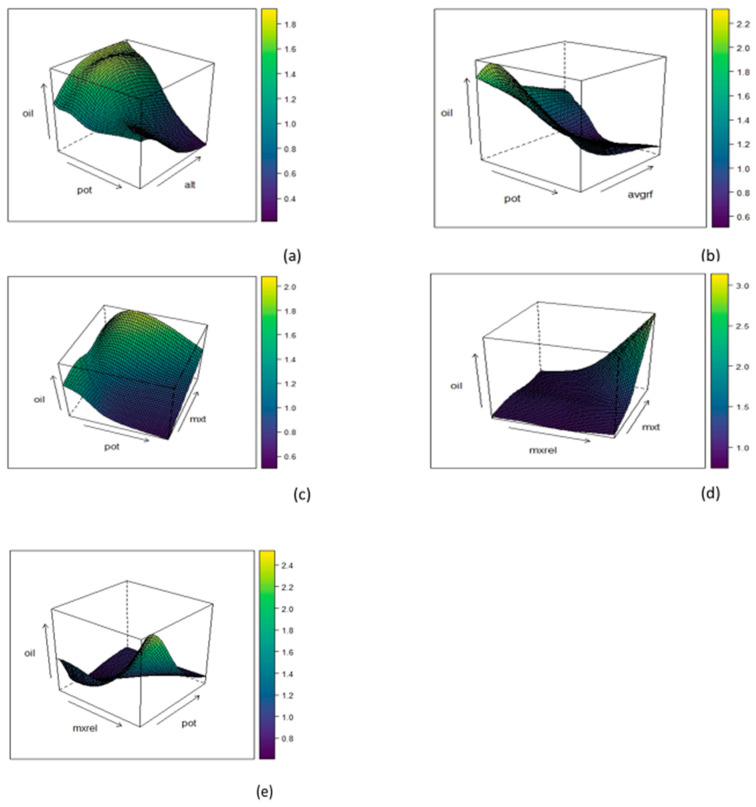
(**a**) 3D partial dependence plot for soil potassium content, altitude, and essential oil content; (**b**) 3D partial dependence plot for soil potassium content, average rainfall, and essential oil content; (**c**) 3D partial dependence plot for soil potassium content, average temperature, and essential oil content; (**d**) 3D partial dependence plot for soil potassium content, average maximum relative humidity, and essential oil content (**e**) 3D partial dependence plot for soil potassium content, pH, and essential oil content.

Partial dependence plots with two variables show the mutual contribution of the variables to the response. Such plots help identify the optimum range of predictor values for a maximum value of the response. Two-variable PDPs (Figure 7a–e and Figure 8a–e) are generated for the top five important variables (soil potassium content, altitude, maximum relative humidity, maximum average temperature, and average rainfall) to understand the mutual influence of these variables on response. For each pair of predictors, two different types of PDPs are generated: a 2D contour plot and 3D partial dependence plot. In each plot, the colour scale on the right-hand side shows the colour as a measure of essential oil content. From these values, it is evident that a lower value of soil potassium content, i.e., from 0 to 250 kg/ha (approx.), and an altitude range of 400 m to 800 m is favorable for higher oil content (Figure 7a and Figure 8a). Similarly, a low value of average rainfall, i.e., 4 mm was found to be favourable for essential oil content (Figure 7b and Figure 8b). The essential oil content is found to be higher when the maximum average temperature value is higher than 36 °C (Figure 7c and Figure 8c). From Figure 7d and Figure 8d, it is evident that a higher maximum relative humidity value (i.e., beyond 95) is found to be favourable for Thai basil oil.

**Figure 9:**
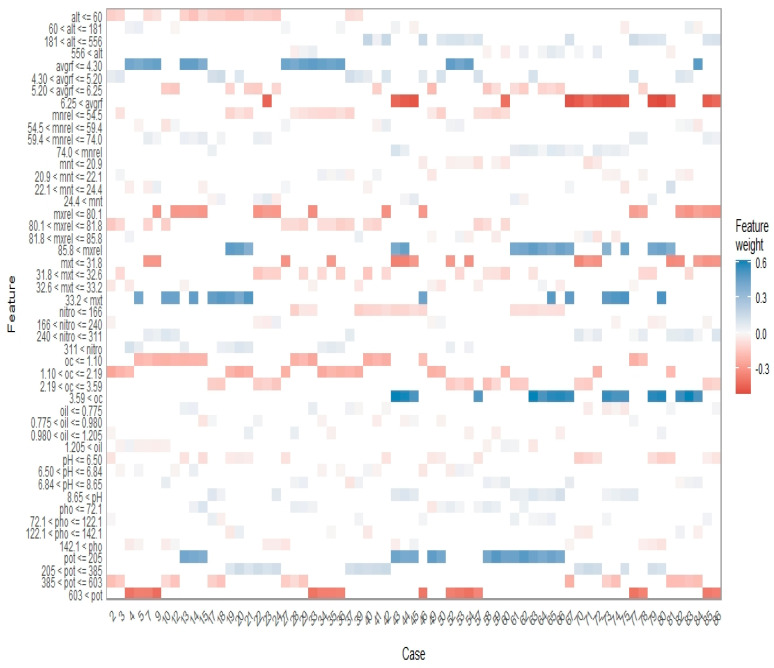
Faceted heatmap showing effect of various predictor value ranges on response.

In the case of all previous PDPs (single-variable and double-variable PDPs), the variation in oil content is represented as a gradual change in colour intensities. From these plots, it is notably challenging to conclude the precise value ranges of variables in which the response shows variation. In such situations, faceted heatmaps are found to be advantageous. A faceted heatmap for all predictors is provided in Figure 9. The colour scale shows the weight or influence of the predictor on response. The *x*-axis shows the instances. The *y*-axis shows the predictor value ranges. The dark blue colour represents positive feature weight, i.e., the variable range supports the response and the dark red color represents negative feature weight [46]. In the plot, the total altitude range is divided into four different groups: ≤60 kg/ha, between 60 kg/ha and 181 kg/ha, 181 to 556 kg/ha, and greater than 556 kg/ha. The first group, ≤60 kg/ha, has a mild negative feature weight; the third group, 181 to 556 kg/ha, has a mild positive feature weight. Average rainfall higher than 6.25 mm has a strong negative effect on basil oil content. The oil content is favoured when the potassium content is less than or equal to 205 kg/ha. Potassium content greater than 603 kg/ha is found to have a negative effect on the oil content. The oil content is found to be positively affected when the maximum relative humidity value is beyond 85.8. The feature weights for other variables are provided in Figure 9. Large multivariate data sets can be explored using heat maps. Color gradients or colour schemes are used to illustrate response variables. The right transformation along with row and column grouping might reveal interesting patterns in the data. In addition, they can be used to display the outcomes of statistical analysis, such as the variables that differ between treatment groups.

To the best of our knowledge, this research is the first to look into the impact of soil nutritional parameters and environmental factors on Thai basil oil content in 10 different agroclimatic regions of Odisha. According to this study, a combination of either two factors or more than two factors have greater impacts than a single factor on Thai basil oil content. It was found that adjusting parameters such as height, temperature, rainfall, humidity, and potassium content in the soil can maximize the oil content of Thai basil. The difference in oil content in different regions is influenced by various parameters that need to be further investigated. The prediction model developed in this study will be beneficial to obtain Thai basil oil content in a new site that is close to the experimental values.

## 4. Conclusions

To obtain the highest oil yield from Thai basil, it will be advantageous to use the artificial neural network (ANN) model developed to investigate the effect of various environmental factors and soil parameters on oil content. The outcome showed that by developing an ANN, we can forecast the oil concentration at a new site using a combination of environmental and soil data. By modifying the model’s input parameters (height, temperature, rainfall, humidity, and potassium), it is possible to increase the oil content of Thai basil. The ANN model is therefore highly helpful to predict and optimize the oil content of Thai basil at a particular site for commercial cultivation.

## Figures and Tables

**Table 1:** Geographic locations and habitat characteristics of Thai basil.

SL. No.	Agroclimatic Zones	Districts	Accession No.	Latitude	Longitude	Altitude
1.	East and South East Coastal Plain	Jagatsingpur	T1	20.2549° N	86.1706° E	46
			T2	20.2553° N	86.1735° E	43
			T3	20.2555° N	86.1740° E	41
		Khurda	T4	20.1869° N	86.1737° E	75
			T5	20.1863° N	85.6223° E	181
			T6	20.1850° N	85.6215° E	195
		Puri	T7	20.1868° N	85.6234° E	0.1
			T8	19.8135° N	85.8312° E	74
			T9	19.8120° N	85.8340° E	85
		Nayagarh	T10	19.8142° N	85.8318° E	178
			T11	20.1231° N	85.1038° E	14
			T12	20.1251° N	85.1045° E	55
2.	North Eastern Coastal Plain	Bhadrak	T13	21.0574° N	86.4963° E	23
			T14	21.0570° N	86.4968° E	23.6
			T15	21.0566° N	86.4970° E	24
		Balasore	T16	21.4934° N	86.9135° E	16
			T17	21.4940° N	86.9140° E	16.3
			T18	21.4944° N	86.9145° E	17
		Jajpur	T19	20.8341° N	86.3326° E	8
			T20	20.8348° N	86.3330° E	9
			T21	20.8352° N	86.3338° E	10
3.	North Eastern Ghat	Ganjam	T22	19.3874° N	85.0515° E	3
			T23	19.3870° N	85.0520° E	568
			T24	19.3866° N	85.0525° E	570
		Gajapati	T25	19.1912° N	84.1857° E	180.5
			T26	19.1918° N	84.1860° E	180.7
			T27	19.1924° N	84.1863° E	181
		Kandhamal	T28	20.1342° N	84.0167° E	700
			T29	20.1348° N	84.0170° E	591
			T30	20.1354° N	84.0173° E	550
4.	Mid Central Table Land	Angul	T31	20.8444° N	85.1511° E	876
			T32	20.8450° N	85.1520° E	218.3
			T33	20.8456° N	85.1526° E	224
		Dhenkanal	T34	20.6505° N	85.5981° E	80
			T35	20.6510° N	85.5988° E	79.6
			T36	20.6515° N	85.5995° E	79
		Cuttack	T37	20.4625° N	85.8830° E	36
			T38	20.4630° N	85.8840° E	36
			T39	20.4635° N	85.8850° E	37
5.	Western Central Table Land	Boudh	T40	20.8418° N	84.3200° E	218
			T41	20.8420° N	84.3202° E	221
			T42	20.8422° N	84.3204° E	226
		Bargarh	T43	21.3470° N	83.6320° E	171
			T44	21.3472° N	83.6322° E	170
			T45	21.3474° N	83.6324° E	169
		Jharsuguda	T46	21.8554° N	84.0062° E	218
			T47	21.8562° N	84.0065° E	216
			T48	21.8560° N	84.0065° E	214
6.	Eastern Ghat High Land	Nawarangpur	T49	19.2281° N	82.5470° E	557
			T50	19.2288° N	82.5478° E	553
			T51	19.2295° N	82.5484° E	548
		Rayagada	T52	19.1712° N	83.4163° E	207
			T53	19.1718° N	83.4169° E	217
			T54	19.1724° N	83.4174° E	227
		Koraput (East)	T55	18.8561° N	82.7347° E	218
			T56	18.8570° N	82.7355° E	218
			T57	18.8579° N	82.7362° E	219
7.	North Central Plateau	Mayurbhanj (South)	T58	22.0087° N	86.4187° E	559
			T59	22.0090° N	86.4193° E	564
			T60	22.0093° N	86.4197° E	568
		Keonjhar (North)	T61	21.6289° N	85.5817° E	596
			T62	21.6287° N	85.5815° E	593
			T63	21.6285° N	85.5813° E	590
		Mayurbhanj (North)	T64	22.0087° N	86.4187° E	570
			T65	22.0091° N	86.4196° E	596
			T66	22.0095° N	86.4205° E	610
8.	South Eastern Ghat	Keonjhar (South)	T67	21.6289° N	85.5817° E	193
			T68	21.6285° N	85.5813° E	193
			T69	21.6281° N	85.5810° E	193
		Koraput (South-East)	T70	18.8561° N	82.7347° E	870
			T71	18.8566° N	82.7354° E	356
			T72	18.8572° N	82.7359° E	110
		Malkangiri	T73	18.3436° N	81.8825° E	178
			T74	18.3441° N	81.8821° E	170
			T75	18.349° N	81.8817° E	162
9.	North Western Plateau	Sundargarh	T76	22.1240° N	84.0432° E	233
			T77	22.1248° N	84.0437° E	231
			T78	22.1256° N	84.0442° E	229
		Deogarh	T79	21.5383° N	84.7289° E	254
			T80	21.5388° N	84.7293° E	253
			T81	21.5392° N	84.7297° E	252
		Sambalpur	T82	21.4669° N	83.9812° E	135
			T83	21.4673° N	83.9818° E	252
			T84	21.4677° N	83.9822° E	312
10.	Western Undulating Zone	Kalahandi	T85	19.9137° N	83.1649° E	355
			T86	19.9141° N	83.1653° E	352
			T87	19.9146° N	83.1657° E	349
		Bolangir	T88	20.7011° N	83.4846° E	383
			T89	20.7017° N	83.4848° E	556
			T90	20.7023° N	83.4850° E	615
		Nuapada	T91	20.8060° N	82.5361° E	1200
			T92	20.8068° N	82.5368° E	1202
			T93	20.8076° N	82.5375° E	1204

**Table 2:** Climatic data for Thai basil from different agroclimatic regions of Odisha.

SL. No.	Agroclimatic Zones	Districts	Accession No.	pH	Max. Rel. Humidity	Min. Rel. Humidity	Avg. Rainfall	Max. Avg. Temp.	Min. Avg. Temp.	Altitude
1.	East and South East Coastal Plain	Jagatsingpur	T1	6.5	81.7	54.3	4.8	32.9	21.9	46
			T2	6.4	81.2	55.1	4.6	32.4	21.5	43
			T3	6.3	81	55	4.5	32.2	21.2	40
		Khurda	T4	6.74	82.1	56.8	3.2	32.8	22.8	75
			T5	6.9	81	59.7	5.4	33.9	21.6	181
			T6	6.99	80.98	59.9	5.9	4.1	21.1	190
		Puri	T7	6.84	80.2	62.9	3.4	30.6	23.4	0.1
			T8	8.4	75.3	71.6	6.4	34	24.1	74
			T9	8.9	72.2	73.4	7.2	35.6	25.4	86
		Nayagarh	T10	7	81.3	59.4	5.6	33.5	21.7	178
			T11	5.1	100.3	25.1	4.9	39.2	12.1	14
			T12	5	102.3	20.8	4.3	41.2	10.5	55
2.	North Eastern Coastal Plain	Bhadrak	T13	6.5	78.2	62.3	3.9	33.1	22.4	23
			T14	6.9	78.6	62.5	3.5	33.2	22.8	23.6
			T15	7.1	79.2	62.9	3.1	33.4	23.2	24
		Balasore	T16	13.7	83	74	4.8	34.2	32.4	16
			T17	13.2	82.8	74.9	4.6	34.6	32.8	16.3
			T18	13.1	82.5	75	4.4	34.9	33.2	16.9
		Jajpur	T19	4.3	98.9	23.9	5.4	38.7	11.4	8
			T20	4.7	98.2	23.1	5.9	38.1	11.7	9
			T21	8	97.9	22.8	6.4	37.7	12.1	10
3.	North Eastern Ghat	Ganjam	T22	8.6	74.6	71.3	6.1	32	27	3
			T23	8.7	87.5	92.8	6.1	33.3	27.4	568
			T24	8.8	90.2	98.9	6.2	33.7	28.1	569
		Gajapati	T25	6.08	80.3	60.1	3.4	30.2	23.8	180.5
			T26	6.21	80.1	59.8	3.5	30.4	23.9	180.7
			T27	6.42	79.8	59.4	3.6	30.6	24	181
		Kandhamal	T28	7.3	80.5	53.8	4.3	32.8	22.1	700
			T29	9.2	97.4	84.3	5.2	32.3	24.4	591
			T30	10.1	106.2	97.8	6.1	31.7	26.2	341
4.	Mid Central Table Land	Angul	T31	7.61	76.2	50.9	3.82	31.2	18.1	876
			T32	6.79	79.7	55.7	5.3	34.2	19.3	218.3
			T33	6.12	81.2	60.1	7.5	37.6	20.2	216.7
		Dhenkanal	T34	6.75	81.4	51.8	4.08	31.8	21.4	80
			T35	6.79	81.9	51.4	4.12	31.6	21.2	79.6
			T36	7.12	82.3	50.9	4.16	31.4	21	79.1
		Cuttack	T37	6.82	81.8	54.3	4.9	33.2	21.9	36
			T38	6.86	81.8	54.3	4.9	33.2	21.9	36
			T39	6.91	82	54.6	5.1	32.6	22.2	36.4
5.	Western Central Table Land	Boudh	T40	6.6	80.7	58.4	5.1	32.5	22.3	218
			T41	6.45	80.9	58.7	4.9	32.1	22.6	221
			T42	6.23	81.1	59.2	4.5	30.9	22.9	225
		Bargarh	T43	10.8	86.7	82.2	7.7	29.3	25.4	171
			T44	11	87.1	82	7.5	29.4	25.6	170
			T45	11.3	87.8	81.9	7.3	29.5	25.9	169
		Jharsuguda	T46	6.8	80.1	55.3	5.1	34.6	19.5	218
			T47	6.45	81	53	4.5	33	19	216
			T48	6.24	81.8	51.5	3.9	32.6	18.5	214
6.	Eastern Ghat High Land	Nawarangpur	T49	6.2	82.4	54.1	5.4	32.7	21.9	557
			T50	6.3	83	54.3	5.5	32.5	21.6	553
			T51	6.4	83.4	54.7	5.9	32.2	21.3	550
		Rayagada	T52	6.83	82.5	54.9	3	31.7	18.9	207
			T53	6.65	81.5	53.6	4.6	32.8	19.1	217
			T54	6.45	80.5	52.4	5	33.3	19.6	227
		Koraput (East)	T55	6.51	81.3	53.1	4.7	33.1	19.2	218
			T56	6.54	81.3	53.1	4.7	33.1	19.2	218
			T57	6.98	81.6	53.8	5.2	33.8	19.6	219
7.	North Central Plateau	Mayurbhanj (South)	T58	7.1	81.7	54.3	6.2	32.1	18.7	559
			T59	7.3	81.4	54.8	6.5	32.9	18.2	564
			T60	7.5	81.1	55.2	6.7	333.3	17.8	569
		Keonjhar (North)	T61	9.2	98	85	5.5	32.8	24.8	596
			T62	9.1	97.6	84.6	5.4	32.5	24.6	593
			T63	9	97.1	84.1	5.3	32.1	24.4	590
		Mayurbhanj (North)	T64	8.9	87.8	93	5.9	33.1	27.6	570
			T65	9.3	98	85	5.5	32.8	24.8	596
			T66	9.6	98.3	80	5.1	32.2	21.2	605
8.	South Eastern Ghat	Keonjhar (South)	T67	7.6	89	93	9.4	38.2	24.2	193
			T68	7.3	89	93	9.4	38.2	24.2	193
			T69	7.9	90	94	9.6	38.4	24.4	194
		Koraput (South-East)	T70	5.8	85	77	8.7	29.6	20.6	870
			T71	4.5	78.4	64.3	8.59	25.8	23.7	356
			T72	4	72.3	57.7	8.34	21.4	26.7	350
		Malkangiri	T73	12.6	86	83	11.9	36	22	178
			T74	10.7	86.2	82.4	7.6	29.5	25.3	170
			T75	8.6	86.4	81.5	6.8	24.8	27.6	162
9.	North Western Plateau	Sundargarh	T76	11.3	66	64	5.4	31.5	20.5	233
			T77	10.9	65.8	64.3	5.2	31.2	20.3	231
			T78	10.5	65.2	64.8	5	30.9	20.1	229
		Deogarh	T79	3.1	97.9	56.1	11	32	25	254
			T80	3.2	98.1	55.9	10.9	31.7	24.7	253
			T81	3.3	98.6	55.2	10.3	31.2	24.2	252
		Sambalpur	T82	7.9	79.4	59.1	4.5	31.3	20.9	135
			T83	3.4	98.1	55.9	10.9	31.8	24.7	252
			T84	2.9	99.3	51.2	16.2	32.5	27.3	255
10.	Western Undulating Zone	Kalahandi	T85	4.4	78	64	8.76	26	24	355
			T86	4.7	77.8	63.8	8.7	25.4	23.6	352
			T87	5.1	77.1	63.2	8.6	24.8	23.2	350
		Bolangir	T88	6.2	76	74	8.01	32.8	30.8	383
			T89	6.3	82.3	53.8	5.3	32.5	21.7	556
			T90	6.5	88.4	51.2	5.1	31.8	21.1	565
		Nuapada	T91	2.1	92	63	9.69	30	23	1200
			T92	2.4	91.7	62.8	9.71	29.8	21	1202
			T93	2.7	91.4	62.2	9.75	29.1	20	1201

**Table 3:** Physicochemical properties of soil samples collected from different agroclimatic regions of Odisha.

SL. No.	Agroclimatic Zones	Districts	Accession No.	Organic Carbon (%)	Nitrogen	Phosphorous	Potassium
1.	East and South East Coastal Plain	Jagatsingpur	T1	1.59	236.2	79.4	542.2
			T2	1.61	235.9	81.4	538.7
			T3	1.63	235.5	81.8	532.5
		Khurda	T4	1.11	503.7	161.7	918.4
			T5	1.41	166.5	217.2	493.2
			T6	1.62	160.7	220.8	490.5
		Puri	T7	0.99	305.31	265.1	796.94
			T8	1.7	270.4	63.4	408.3
			T9	2	255.3	62.5	400.9
		Nayagarh	T10	0.76	250	169.05	491.9
			T11	0.83	250.4	169.02	491.2
			T12	0.91	250.8	169	491
2.	North Eastern Coastal Plain	Bhadrak	T13	0.87	352.5	64.7	201.6
			T14	0.82	352.1	64.2	201.2
			T15	0.78	351.8	63.8	200.8
		Balasore	T16	3.2	340.2	83.2	602.3
			T17	3.4	179.5	281.9	209.4
			T18	3.6	178.9	280.8	208.7
		Jajpur	T19	1.8	375.4	132.2	302.5
			T20	2	381.5	130.3	294.6
			T21	2.2	385.7	128.8	290.2
3.	North Eastern Ghat	Ganjam	T22	3.1	183.7	280.3	208.3
			T23	3.4	340.6	83.8	602.4
			T24	3.6	341.3	82.6	601.1
		Gajapati	T25	1.36	166.2	217.5	493.9
			T26	1.27	176	26.58	78.1
			T27	1.11	186.2	25.6	76.8
		Kandhamal	T28	0.52	141.2	37.2	519
			T29	0.58	141.3	37.5	519.7
			T30	1.02	141,5	37.9	520.2
4.	Mid Central Table Land	Angul	T31	0.94	162.3	33.2	771.6
			T32	0.91	162.7	33.7	771.8
			T33	0.89	163.2	34.1	772.1
		Dhenkanal	T34	1.79	562.5	132.4	921.6
			T35	1.81	562.1	132.1	921.3
			T36	1.86	558.5	131.8	920.8
		Cuttack	T37	1.5	251.2	96.3	306
			T38	3.61	152.4	72.6	89.1
			T39	3.89	150.8	70.9	88.1
5.	Western Central Table Land	Boudh	T40	0.32	125	127.91	309.12
			T41	0.35	123	128.1	310.1
			T42	0.37	121	128.7	310.8
		Bargarh	T43	5.2	140.3	142.3	30
			T44	5.4	140.7	141.9	32
			T45	5.6	141.1	141.4	33
		Jharsuguda	T46	0.94	112.5	75.5	603.46
			T47	0.91	112.4	74.9	602.9
			T48	0.88	112.3	74.4	602.23
6.	Eastern Ghat High Land	Nawarangpur	T49	1.14	175	26.48	77.95
			T50	1.01	113	75.3	603.8
			T51	1	110	97.8	602.4
		Rayagada	T52	3.27	316.2	29.3	924.8
			T53	3.98	164.5	82.4	93.4
			T54	4.02	163.8	84.7	94.6
		Koraput (East)	T55	4.61	381.2	32.4	961.3
			T56	4.74	380.6	32.1	960.8
			T57	4.82	380.3	31.8	960.4
7.	North Central Plateau	Mayurbhanj (South)	T58	2.23	175	50.72	73.92
			T59	4.01	164.3	82.6	93.1
			T60	6.32	160.8	88.9	96.8
		Keonjhar (North)	T61	3.57	152.7	72.8	89.3
			T62	3.65	152.5	72.4	89.1
			T63	3.99	152.3	72	88.8
		Mayurbhanj (North)	T64	3.98	164.5	82.4	93.4
			T65	4.72	383.2	32.7	959.6
			T66	5.88	386.8	31.6	960.6
8.	South Eastern Ghat	Keonjhar (South)	T67	8.4	261	145.2	391.2
			T68	8	231	152	308.7
			T69	7.6	201	165.9	300.7
		Koraput (South-East)	T70	2.3	242.3	132.7	296.3
			T71	3.4	340.4	83.5	602.4
			T72	4.6	444.4	82.9	603.9
		Malkangiri	T73	6.36	216.4	121.2	386.4
			T74	5.1	140.1	142.5	29.8
			T75	5	132.4	162.6	30
9.	North Western Plateau	Sundargarh	T76	1.06	285.3	161.2	603.4
			T77	1.1	285.1	161.4	603.1
			T78	1.4	284.8	161.6	603
		Deogarh	T79	8.1	230.4	152.3	308.5
			T80	8.3	230.5	152.7	308.8
			T81	8.5	230.6	153.1	309.1
		Sambalpur	T82	8.6	262.5	139.75	594.8
			T83	8.9	263.7	139.65	592.6
			T84	9.2	264.1	139.55	590.4
10.	Western Undulating Zone	Kalahandi	T85	2.4	240.3	76.3	813.7
			T86	2.5	240.4	76.2	813.6
			T87	2.6	240.5	76.1	813.5
		Bolangir	T88	8.9	290.4	89.1	503.1
			T89	8	230.2	152.7	308.3
			T90	7.2	229.8	153.8	307.6
		Nuapada	T91	1.6	270.3	63.2	408.1
			T92	1.18	175.4	26.51	77.81
			T93	1.02	174.3	25.65	75.75

**Table 4:** Predicted and actual Thai basil oil content for the train set.

SL. No.	Agroclimatic Zones	Districts	Accession no.	Experimental Thai Basil Oil Yield (X1)	Predicted Thai Basil Oil Yield (X2)	Absolute = |X1 − X2|
1.	East and South East Coastal Plain	Jagatsingpur	T1	1.3	1.29	0.01
		Khurda	T4	1.42	1.42	0
			T5	1.21	1.21	0
			T6	1.2	1.19	0.01
		Puri	T7	1.1	1.11	0.01
			T8	1.65	1.67	0.02
2.	North Eastern Coastal Plain	Bhadrak	T13	0.76	0.76	0
			T14	1.45	1.43	0.02
			T15	0.81	0.79	0.02
		Jajpur	T19	0.4	0.40	0
			T20	0.9	0.88	0.02
			T21	0.98	1.01	0.03
3.	North Eastern Ghat	Ganjam	T22	0.97	0.98	0.01
		Gajapati	T25	0.81	0.77	0.04
			T26	0.94	1	0.06
			T27	1.68	1.68	0
		Kandhamal	T28	1.2	1.20	0
			T29	1.07	1.06	0.01
			T30	1.1	1.08	0.02
4.	Mid Central Table Land	Angul	T31	0.78	0.79	0.01
			T32	1.18	1.18	0
		Dhenkanal	T34	0.98	0.96	0.02
		Cuttack	T37	0.67	0.67	0
			T38	1	1	0
			T39	0.65	0.65	0
5.	Western Central Table Land	Boudh	T40	1.01	0.96	0.05
			T41	0.78	0.80	0.02
		Bargarh	T43	0.89	0.90	0.01
			T44	0.98	0.98	0
		Jharsuguda	T46	0.92	0.92	0
			T47	0.76	0.75	0.01
6.	Eastern Ghat High Land	Nawarangpur	T49	0.73	0.73	0
			T50	0.76	0.78	0.02
			T51	1.27	1.24	0.03
		Rayagada	T52	0.9	0.90	0
			T53	0.95	1	0.05
			T54	0.97	0.98	0.01
		Koraput (East)	T55	0.91	0.91	0
			T56	0.93	0.95	0.02
			T57	0.69	0.67	0.02
7.	North Central Plateau	Mayurbhanj (South)	T58	1.4	1.38	0.02
			T59	1.2	1.25	0.05
			T60	1.3	1.32	0.02
		Keonjhar (North)	T61	3.62	3.57	0.13
			T62	3.5	3.52	0.02
		Mayurbhanj (North)	T64	0.93	0.98	0.05
			T65	0.91	0.96	0.05
			T66	1.1	1.07	0.03
8.	South Eastern Ghat	Keonjhar (South)	T67	3.94	3.94	0
			T68	3.7	3.73	0.03
		Koraput (South-East)	T70	0.72	0.75	0.03
			T71	1.02	0.97	0.05
			T72	1.3	1.34	0.04
		Malkangiri	T73	0.64	0.61	0.03
			T74	0.76	0.77	0.01
9.	North Western Plateau	Sundargarh	T76	0.68	0.69	0.01
			T77	0.79	0.78	0.01
		Sambalpur	T82	1.1	1.12	0.02
			T83	1.25	1.27	0.02
			T84	1.2	1.20	0
10.	Western Undulating Zone	Kalahandi	T85	0.36	0.36	0
			T86	0.12	0.13	0.01
		Bolangir	T88	1.3	1.30	0
			T89	1.5	1.45	0.05
			T90	1.1	1.09	0.01
		Nuapada	T91	0.7	0.69	0.01
			T92	0.9	0.89	0.01

**Table 5:** Predicted and actual Thai basil oil content for test set.

SL. No.	Agroclimatic Zones	Districts	Accession no.	Experimental Thai Basil Oil Yield (X1)	Predicted Thai Basil Oil Yield (X2)	Absolute = |X1 − X2|
1.	East and South East Coastal Plain	Jagatsingpur	T2	1.2	1.19	0.01
			T3	1.2	1.27	0.07
		Nayagarh	T10	1.3	1.27	0.030
			T11	0.65	0.77	0.12
			T12	1.39	1.41	0.02
2.	North Eastern Coastal Plain	Balasore	T16	0.81	0.84	0.03
			T17	1	1.29	0.29
			T18	1	1.28	0.28
3.	North Eastern Ghat	Ganjam	T23	0.94	0.94	0
4.	Mid Central Table Land	Dhenkanal	T34	0.75	0.78	0.03
			T35	0.69	0.73	0.04
5.	Western Central TableLand	Boudh	T42	0.86	0.90	0.04
6.	North Central Plateau	Keonjhar (North)	T61	3.72	3.16	0.56
7.	South Eastern Ghat	Keonjhar (South)	T67	4.1	4.46	0.36
		Malkangiri	T75	0.71	0.66	0.05
8.	North Western Plateau	Sundargarh	T78	0.95	0.96	0.01
		Deogarh	T79	1	1.28	0.28
			T80	0.75	0.78	0.03
			T81	0.38	0.34	0.04
9.	Western Undulating Zone	Kalahandi	T87	0.78	0.53	0.25

**Table 6:** Predicted and actual Thai basil content for validation set.

SL. No.	Agroclimatic Zones	Districts	Accession No.	Experimental Thai Basil Oil Yield (X1)	Predicted Thai Basil Oil Yield (X2)	Absolute = |X1 − X2|
1.	East and South East Coastal Plain	Puri	T9	0.13	0.14	0.01
2.	North Eastern Ghat	Ganjam	T24	0.94	0.97	0.03
3.	Mid Central Table Land	Angul	T33	0.78	0.60	0.18
4.	Western Central Table Land	Bargarh	T45	0.8	0.69	0.11
		Jharsuguda	T48	0.77	0.55	0.22
5.	Western Undulating Zone	Nuapada	T93	0.13	0.13	0

**Table 7:** Sensitivity values of different factors affecting oil production of Thai basil.

Predictors	Sensitivity Value
pH	2.32
Organic Carbon	2.31
Nitrogen	109.99
Phosphorous	65.47
Potassium	284.49
Maximum relative humidity	6.62
Minimum relative humidity	14.42
Average rainfall	2.21
Maximum average temperature	2.46
Minimum average temperature	3.66
Altitude	274.38

## Data Availability

The study did not report any data.

## Appendix A. Sample Code

library(readxl)
library(caret)
data<-as.data.frame(read_xlsx(“tb.xlsx”))
#calculate standard deviation
library(mlbench)
library(“openxlsx”)
# calculate standard deviation for all attributes
sd.table<-sapply(data[,1:12], sd)
sd.table<-as.data.frame(sd.table)
write.xlsx(sd.table,”Standarddeviation.xlsx”)
######Correlations analysis
correlations <- cor(data[,1:10])
# display the correlation matrix
cor.table<-print(correlations)
cor.table
write.xlsx(cor.table,”Correlations.xlsx”)
#creating panel plot
library(psych)
pairs.panels(data[c(“pH”, “oc”, “nitro”, “pho”,”pot”,”mxrel”,”mnrel”,”avgrf”,”mxt”,”mnt”,”alt”)])
#############data splitting ###########
set.seed(212)
trainIndex<- createDataPartition(data$oil, p=0.8, list=FALSE)
train <- data[ trainIndex,] ####80% of total data including validation
test<- data[-trainIndex,]
validIndex<- createDataPartition(data$oil, p=0.9, list=FALSE)
train <- data[ validIndex,] ####70% of total data
valid<- data[-validIndex,] ######10% of total data
#######ANN model development######
trainControl <- trainControl(method=“cv”, number=15)
tunegrid<- expand.grid(layer1 = 16, layer2 =15, layer3=4)
metric <- “MAE”
set.seed(123)
fit.ann<-train(oil~., data = train, method=“neuralnet”, metric = metric, preProcess=c(“range”), tuneGrid = tunegrid, act.fct=“logistic”,trControl = trainControl,learningrate = 0.02, linear.output=T)
fit.ann
####Plotting the final model#####
library(NeuralNetTools)
plotnet(fit.ann$finalModel)
####Predicting for test set####
pr.ann <- predict(fit.ann, newdata=test)
pr.ann<-as.data.frame(pr.ann)
library(“openxlsx”)
output.table<-cbind(test$oil,pr.ann)
output.table
write.xlsx(output.table,’testVsPred.xlsx’)
#####Predicting for validation set
valid<-as.data.frame(read_xlsx(“valid.xlsx”))
val.ann <- predict(fit.ann, newdata=valid)
val.ann<-as.data.frame(val.ann)
########Variable Importance
library(NeuralNetTools)
olden(fit.ann)
#####partial dependecnce plot
#####Single variable
library(pdp)
library(ggplot2)
partial(pred.var = “alt”) %>%
plotPartial(smooth = T, lwd = 2, ylab = expression(f(alt)))
partial(pred.var = “mnt”) %>%
plotPartial(smooth = T, lwd = 2, ylab = expression(f(mnt)))
partial(pred.var = “mxt”) %>%
plotPartial(smooth = T, lwd = 2, ylab = expression(f(mxt)))
partial(pred.var = “mxrel”) %>%
plotPartial(smooth = T, lwd = 2, ylab = expression(f(mxrelhum)))
partial(pred.var = “avgrf”) %>%
plotPartial(smooth = T, lwd = 2, ylab = expression(f(avgrainfall)))
partial(pred.var = “mnrel”) %>%
plotPartial(smooth = T, lwd = 2, ylab = expression(f(mnrel)))
partial(pred.var = “pot”) %>%
plotPartial(smooth = T, lwd = 2, ylab = expression(f(pot)))
partial(pred.var = “pho”) %>%
plotPartial(smooth = T, lwd = 2, ylab = expression(f(pho)))
partial(pred.var = “nitro”) %>%
plotPartial(smooth = T, lwd = 2, ylab = expression(f(nitro)))
partial(pred.var = “oc”) %>%
plotPartial(smooth = T, lwd = 2, ylab = expression(f(oc)))
partial(pred.var = “pH”) %>%
plotPartial(smooth = T, lwd = 2, ylab = expression(f(pH)))
####without smoothing
fit.ann %>% # the %>% operator is read as “and then”
partial(pred.var = “alt”) %>%
plotPartial(smooth = F, lwd = 2, ylab = expression(f(alt)))
######with two predictors#######
######pot mxrel
par.mxrel.pot <- partial(fit.ann, pred.var = c(“mxrel”, “pot”))
plotPartial(par.mxrel.pot)
# Add contour lines and use a different color palette
rwb <- colorRampPalette(c(“red”, “white”, “blue”))
pdp2 <- plotPartial(par.mxrel.pot, contour = TRUE, col.regions = rwb)
pdp2
# 3-D surface
pdp3 <- plotPartial(par.mxrel.pot,rug=T,levelplot = FALSE,number=4, zlab = “oil”, drape = TRUE,colorkey = TRUE, screen = list(z =−40, x = −60))
pdp3
###Maximum rel humidity and Max. avg. temperature
par.mxrel.mxt <- partial(fit.ann, pred.var = c(“mxrel”, “mxt”))
plotPartial(par.mxrel.mxt)
# Add contour lines and use a different color palette
rwb <- colorRampPalette(c(“red”, “white”, “blue”))
pdp.mxrel.mxt.2d <- plotPartial(par.mxrel.mxt, contour = TRUE, col.regions = rwb)
pdp.mxrel.mxt.2d
# 3-D surface
pdp.mxrel.mxt.3d <- plotPartial(par.mxrel.mxt,rug=T,levelplot = FALSE,number=4, zlab = “oil”, drape = TRUE,colorkey = TRUE, screen = list(z = −20, x = −60))
pdp.mxrel.mxt.3d
###pot mxt
par.pot.mxt <- partial(fit.ann, pred.var = c(“pot”, “mxt”))
plotPartial(par.pot.mxt)
# Add contour lines and use a different color palette
rwb <- colorRampPalette(c(“red”, “white”, “blue”))
pdp.pot.mxt.2d <- plotPartial(par.pot.mxt, contour = TRUE, col.regions = rwb)
pdp.pot.mxt.2d
# 3-D surface
pdp.pot.mxt.3d <- plotPartial(par.pot.mxt,rug=T,levelplot = FALSE,number=4, zlab = “oil”, drape = TRUE,colorkey = TRUE, screen = list(z = −20, x = −40))
pdp.pot.mxt.3d
######pot avgrf
par.pot.avgrf <- partial(fit.ann, pred.var = c(“pot”, “avgrf”))
plotPartial(par.pot.avgrf)
# Add contour lines and use a different color palette
rwb <- colorRampPalette(c(“red”, “white”, “blue”))
pdp.pot.avgrf.2d <- plotPartial(par.pot.avgrf, contour = TRUE, col.regions = rwb)
pdp.pot.avgrf.2d
# 3-D surface
pdp.pot.avgrf.3d <- plotPartial(par.pot.avgrf,rug=T,levelplot = FALSE,number=4, zlab = “oil”, drape = TRUE,colorkey = TRUE, screen = list(z = −40, x = −70))
pdp.pot.avgrf.3d
######pot alt
par.pot.alt <- partial(fit.ann, pred.var = c(“pot”, “alt”))
plotPartial(par.pot.alt)
# Add contour lines and use a different color palette
rwb <- colorRampPalette(c(“red”, “white”, “blue”))
pdp.pot.alt.2d <- plotPartial(par.pot.alt, contour = TRUE, col.regions = rwb)
pdp.pot.alt.2d
# 3-D surface
pdp.pot.alt.3d <- plotPartial(par.pot.alt,rug=T,levelplot = FALSE,number=4, zlab = “oil”, drape = TRUE,colorkey = TRUE, screen = list(z = −40, x = −60))
pdp.pot.alt.3d
##### Faceted heatmap#########
library(lime)
lime.fit<-lime(train,fit.ann)
exp.fit<-explain(train,lime.fit,n_features=10)
plot_explanations(exp.fit)
plot_features(exp.fit)
